# Supplementary material for: EnzML: multi-label prediction of enzyme classes using InterPro signatures
Source: BMC Bioinformatics. 2012 Apr 25;13:61. doi: 10.1186/1471-2105-13-61 (PMC3483700; doi:10.1186/1471-2105-13-61)
Supplement: Addtional file 5 — The Java code to format the data files, evaluate and predict. The file enzml_java_code.tar.gz contains the Java code used to format database data to ARFF and XML formats, to execute cross and train-test (jackknife) evaluations and to record evaluation results to database. More information is included in the readme.txt file and the Javadoc files. The code can be used with a MySQL database. To use a different database software, other JDBC drivers might be required. [file 1471-2105-13-61-S5.gz › java_code/utils/doc/uk/ac/ed/inf/utils/webutils/simpledomparser/XmlNode.html]

XmlNode


---


|  |  |  |
| --- | --- | --- |
| **Overview** \| **Package** \| **Class** \| **Use** \| **Tree** \| **Deprecated** \| **Index** \| **Help** | |  |
| **PREV CLASS**   **NEXT CLASS** | **FRAMES**    **NO FRAMES**     **All Classes** |  |
| SUMMARY: NESTED \| FIELD \| CONSTR \| METHOD | DETAIL: FIELD \| CONSTR \| METHOD |  |


---


## uk.ac.ed.inf.utils.webutils.simpledomparser Class XmlNode

```
java.lang.Object
  uk.ac.ed.inf.utils.webutils.simpledomparser.XmlNode
```

---

``` public class XmlNode extends java.lang.Object ```

`SimpleElement` is the only node type for simplified DOM model.
Adapted from: http://www.devx.com/xml/Article/10114 by Guang Yang

**Version:**
:   12 Dec 2007

**Author:**
:   Luna De Ferrari luna.deferrari-at-ed.ac.uk (adapted from Guang Yang)

---

**Constructor Summary**

`XmlNode(java.lang.String tagName)`


| **Method Summary** | |
| --- | --- |
| `XmlNode` | `addChildNode(XmlNode element)`             Adds a child element to the node, and returns the node itself |
| `java.lang.String` | `getAttributeValue(java.lang.String attributeName)`             Get the attribute value for the given attribute name. |
| `java.util.LinkedList<XmlNode>` | `getChildElements()`             public Object[] getChildElements() { return m\_childElements.toArray(); } |
| `java.lang.String` | `getTagName()`             Get the tag E.g. |
| `java.lang.String` | `getText()`             Get text for the tag E.g. |
| `static XmlNode` | `getXmlTreeFromString(java.lang.String XMLText)` |
| `static XmlNode` | `getXmlTreeFromUrl(java.lang.String url, boolean gzipped)`             get XML tree from URL |
| `boolean` | `hasChildren()`             Whether a SimpleElement node has child nodes. |
| `java.lang.String` | `nodeHeadToString()`             String representation of the node opening tag |
| `void` | `removeAttribute(java.lang.String name)`             remove an attribute by name |
| `XmlNode` | `removeChildNode(XmlNode node)`             Remove a child element |
| `void` | `setAttribute(java.lang.String name, java.lang.String value)` |
| `void` | `setTagName(java.lang.String tagName)` |
| `void` | `setText(java.lang.String text)` |
| `java.lang.String` | `toString()`             Full string representation of node and all child nodes. |

**Methods inherited from class java.lang.Object**

`equals, getClass, hashCode, notify, notifyAll, wait, wait, wait`

**Constructor Detail**

### XmlNode

```
public XmlNode(java.lang.String tagName)
```


**Method Detail**

### addChildNode

```
public XmlNode addChildNode(XmlNode element)
```

:   Adds a child element to the node, and returns the node itself

    :   **Parameters:**: `element` - the child node to be added **Returns:**: the node itself, now containing also the child node

---


### getAttributeValue

```
public java.lang.String getAttributeValue(java.lang.String attributeName)
```

:   Get the attribute value for the given attribute name. E.g. for a
    SimpleElement 0<\/Item>
    this.getAttribute("Name") would return "Status"

    :   **Parameters:**: `attributeName` - the attribute name **Returns:**: the attribute value for the attribute name.

---


### getChildElements

```
public java.util.LinkedList<XmlNode> getChildElements()
```

:   public Object[] getChildElements() { return m\_childElements.toArray(); }

---


### getTagName

```
public java.lang.String getTagName()
```

:   Get the tag E.g. for a SimpleElement 0<\/Item> this.getTag() would return "Item"

    :   **Returns:**: the XML tag name

---


### getText

```
public java.lang.String getText()
```

:   Get text for the tag E.g. for a SimpleElement <Item Name="Status"
    Type="Integer">0</Item> this.getText() would return "0"

    :   **Returns:**: the text value in between the tag

---


### hasChildren

```
public boolean hasChildren()
```

:   Whether a SimpleElement node has child nodes.

    :   **Returns:**: true if the SimpleElement has children nodes.

---


### nodeHeadToString

```
public java.lang.String nodeHeadToString()
```

:   String representation of the node opening tag

    :   **Returns:**

---


### removeAttribute

```
public void removeAttribute(java.lang.String name)
```

:   remove an attribute by name

    :   **Parameters:**: `name` -

---


### removeChildNode

```
public XmlNode removeChildNode(XmlNode node)
```

:   Remove a child element

    :   **Parameters:**: `node` - a child node **Returns:**

---


### setAttribute

```
public void setAttribute(java.lang.String name,
                         java.lang.String value)
```

---


### setTagName

```
public void setTagName(java.lang.String tagName)
```

---


### setText

```
public void setText(java.lang.String text)
```

---


### toString

```
public java.lang.String toString()
```

:   Full string representation of node and all child nodes.

    :   **Overrides:**: `toString` in class `java.lang.Object`

---


### getXmlTreeFromString

```
public static XmlNode getXmlTreeFromString(java.lang.String XMLText)
```

---


### getXmlTreeFromUrl

```
public static XmlNode getXmlTreeFromUrl(java.lang.String url,
                                        boolean gzipped)
```

:   get XML tree from URL

    :   **Parameters:**: `url` -: `gzipped` - whether the url has to be gunzipped **Returns:**: and XML tree


---


|  |  |  |
| --- | --- | --- |
| **Overview** \| **Package** \| **Class** \| **Use** \| **Tree** \| **Deprecated** \| **Index** \| **Help** | |  |
| **PREV CLASS**   **NEXT CLASS** | **FRAMES**    **NO FRAMES**     **All Classes** |  |
| SUMMARY: NESTED \| FIELD \| CONSTR \| METHOD | DETAIL: FIELD \| CONSTR \| METHOD |  |


---
